# Assessing genomics confidence and learning needs in Australian nurses and midwives: an educational program evaluation

**DOI:** 10.3389/fgene.2024.1419302

**Published:** 2024-07-16

**Authors:** Kim E. Alexander, Melanie Rolfe, Michael T. Gabbett

**Affiliations:** ^1^ Centre for Healthcare Transformation, School of Nursing, Queensland University of Technology, Brisbane, QLD, Australia; ^2^ Cancer and Palliative Care Outcomes Centre, School of Nursing, Queensland University of Technology, Brisbane, QLD, Australia; ^3^ Centre for Genomics and Personalised Health, School of Biomedical Sciences, Queensland University of Technology, Brisbane, QLD, Australia

**Keywords:** genomics, nursing, midwifery, professional education, human genetics, health education, learning needs, confidence

## Abstract

**Introduction:** The mainstreaming of genomics across healthcare specialties necessitates that all nurses and midwives have a high literacy in genomics.

**Methods:** We aimed to design, develop, implement and evaluate a genomics education workshop for nurses and midwives using action research principles.

**Results:** Registered nurses and midwives completed an online survey regarding genomics confidence and learning needs (n = 274). The results of this survey were used to develop the genomics education workshop. The workshop was run three times (n = 105) with evaluation data being collected both before and after each workshop. Significant improvements in confidence across all learning domains was found following the workshops (*p* < 0.001). A desire for more education across all learning domains except for genetics knowledge was also identified (*p* < 0.001).

**Discussion:** Genomics education workshops were found to increase the confidence of nurses and midwives across a range of specialties. Nurses and midwives also expressed a desire for further education in genomics.

## 1 Introduction

Timely identification of genomic healthcare needs and accessible services is pivotal to ensuring genomic information is helpful for diagnosis and treatment decisions (Stark et al., 2018). It is also associated with reduced financial and personal costs (Goranitis et al., 2022). Nurses are often a primary point of contact for a person navigating the health system. Therefore, nurses need to be equipped to screen and assess people for genomic needs to facilitate their care (Barr et al., 2018). To ensure the genomic needs of our community are identified and promptly met, there is increasing demand for genomics knowledge and expertise across a range of nursing roles (Lynch et al., 2021).

Poor genomics literacy among nurses and midwives has previously been identified (Wright et al., 2019; White et al., 2020). One contributing factor in Australia is the scant inclusion of genomics education in the undergraduate curricula (Birks et al., 2015). Furthermore, post-qualification programmes are generally not nursing specific, or focus on specialist areas of nursing practice (e.g., oncology) (Paneque et al., 2016; Talwar et al., 2017). Genomics education does not accommodate the needs of most generalist nurses who may work with people with a range of genomic issues. Further, a recent literature review concluded that nursing research must move to interventional studies that integrate genomics into nursing practice (Thomas et al., 2023).

To address this need, we designed, implemented and evaluated a series of genomics education workshops for nurses and midwives working in a variety of settings. To our knowledge, this is the first Australian study to assess the confidence and learning needs of nurses and midwives in genomics before and after genomics education workshops.

## 2 Materials and methods

**FIGURE 1:**
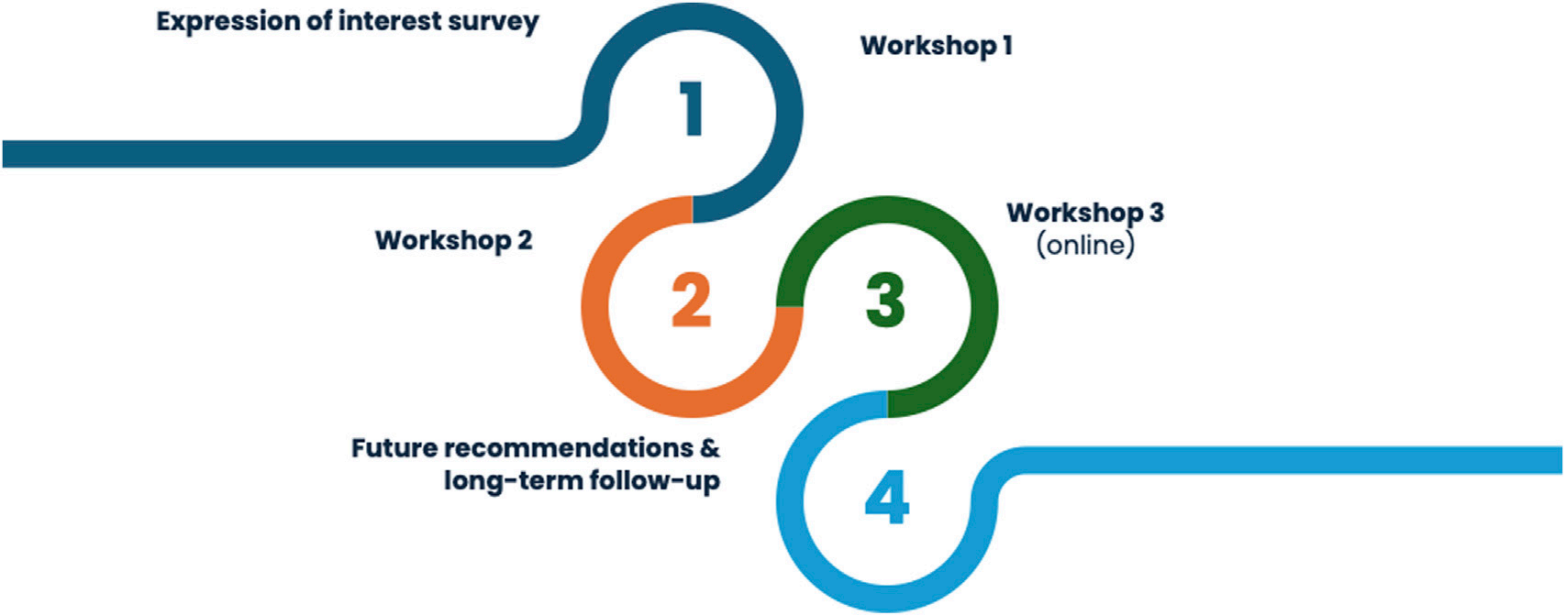
Overview of Workshop Development and Evaluation.

The project utilised Lewin’s action research principles (Lewin, 1946), which follows an iterative process of defining the need, planning and implementing a course of action in response to the need, evaluating the outcomes of that action, and amending subsequent actions based on this evaluation (Figure 1).

### 2.1 Ethics approval

Ethics approval for this study was granted through Queensland University of Technology Human Research Ethics Committee (reference: 2000000827).

### 2.2 Formative research

A survey was used to identify nurses’ and midwives’ current levels of confidence and learning needs regarding genomics. As the authors’ were unable to identify an existing survey instrument that was suitable to the Australian setting, a tool was developed based on literature searches of work that assessed confidence associated with genomics nursing competencies and genomic learning needs for nurses and midwives (Calzone et al., 2012; Greco et al., 2012; Kirk et al., 2014; Saleh et al., 2019; McClaren et al., 2020b). International nursing or midwifery genomic competency standards were also reviewed (Nursing and Midwifery Council, 2018; American Association of Colleges of Nursing, 2020). The items in the survey were piloted with a small convenience sample of nurses and content experts and only typographical changes were made. A review of results identified that an additional two questions were needed that asked about confidence in understanding relevant genetic tests and technologies and experience and learning needs related to genetics.

#### 2.2.1 Survey respondents

The final electronic survey was sent by email to nurses and midwives across the 16 hospitals and health services in Queensland (Supplementary Material). One reminder email was sent out. Survey results informed the development of the first workshop.

### 2.3 Workshops

#### 2.3.1 Workshop development

**FIGURE 2:**
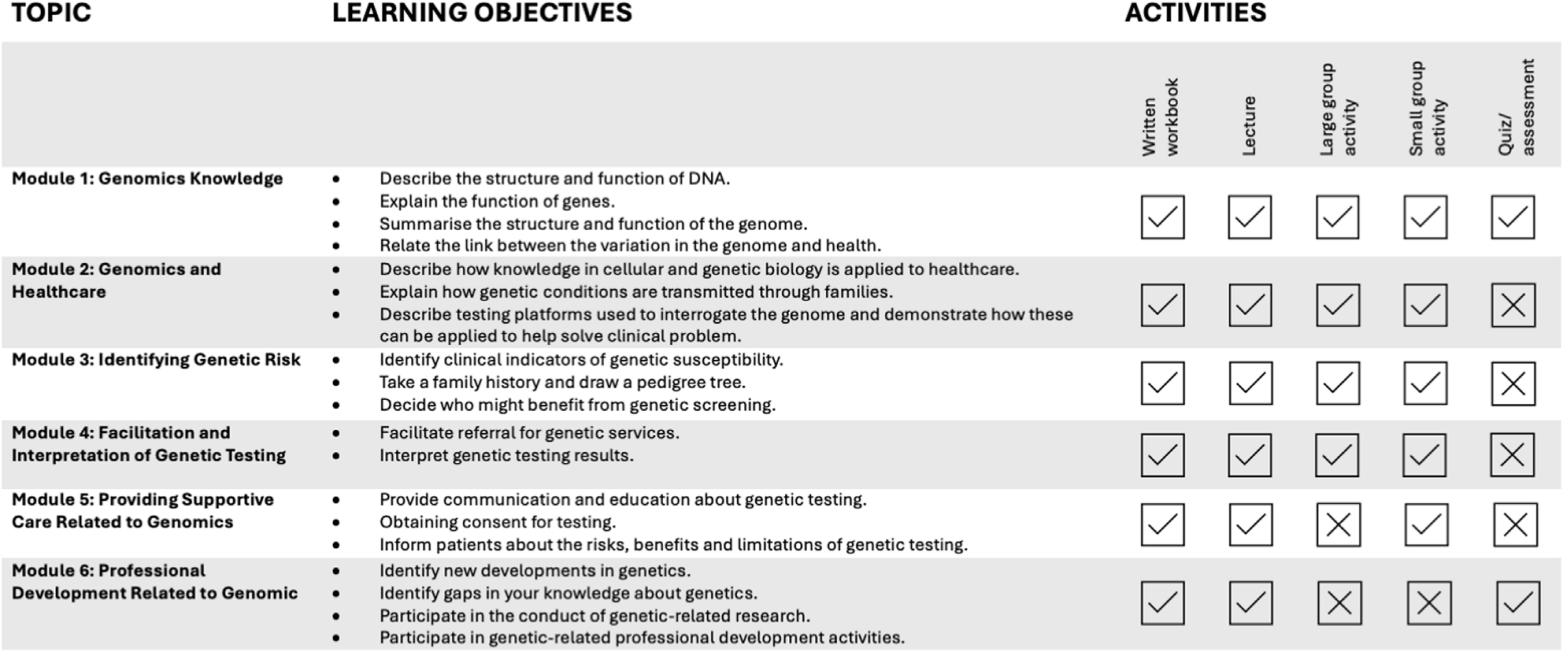
Overview of Workshop Content.

The workshops were informed by the results of the survey and covered topics related to genetics knowledge, genomics in healthcare, identifying genetic risk, facilitation and interpretation of genetic testing, providing supportive care, and professional development related to genetics. The learning objectives aimed to increase genetics practice confidence for each topic (Figure 2). The course development team included a nursing genomics researcher (KA), and a clinical geneticist (MG), both with expertise in higher education of healthcare professionals.

#### 2.3.2 Participants

Survey respondents were invited to the first workshops if they had expressed an interest. Participants were required to be currently registered as a nurse or midwife in Queensland. The workshops were organised by participants’ experience in genomics, with the first workshop open to those with experience in applying genomics knowledge to their practice. This approach allowed the researchers to capture feedback from those with practical experience in applying the knowledge. It also allowed case studies given by participants to be incorporated into subsequent workshops. The remaining two workshops were open to any nurse or midwife.

#### 2.3.3 Workshop delivery

The first two workshops were delivered as face-to-face events over 1 day. These were catered, and some participants were supported to attend in worktime by their supervisors. Workshop three was delivered online, as two workshops of 2 hours, 1 week apart. This was followed by a 3-h workshop, 2 weeks later. All workshops were run in 2021.

The workshops used a mixture of delivery methods including didactic instruction, small and whole group discussion, and activities. Participants were provided with a workbook which also contained further reading and practice activities. All workshops were free.

#### 2.3.4 Workshop evaluation

At the conclusion of each workshop, participants received a form asking for feedback on workshop elements, specifically what to stop, what to start, and what to keep doing. This has been shown to be an effective model for qualitative feedback in higher education (Hoon et al., 2014).

Participants’ confidence and learning needs pre- and post-intervention were also compared.

#### 2.3.5 Data analysis

Items were reported descriptively and variables of interest were compared using the McNemar test. All analyses were conducted using SPSS (version 26, IBM, Armonk, NY, USA). Values of *p* < 0.05 were considered statistically significant.

The qualitative feedback was coded as ‘positive’ or ‘negative’ by two researchers (KA and MR) and quantified, in a modified approach to that used by Hoon et al. (2014). The results informed subsequent revisions of the workshop.

The Standards for Quality Improvement Reporting Excellence for Education (SQUIRE-EDU) was used to report findings (Ogrinc et al., 2019) (Supplementary Material).

## 3 Results

### 3.1 Formative research

There were 274 survey respondents, however not everyone answered all questions. Respondents were primarily registered nurses (83%), female (89%) and over 40 years of age (70%) (Supplementary Material). Most came from metropolitan health services (65%) and had been in their current area of practice for less than 10 years (44%).

#### 3.1.1 Preferences

The most helpful modes of education (rated as ‘quite helpful’ or ‘very helpful’) were: ‘a mix of self-study and face-to-face learning’ (81%), ‘a series of smaller genetic education modules/workshops/presentations’ (80%), ‘face-to-face workshops [mixture of presentations and group activities]’ (80%), and ‘online presentations’ (76%). The least preferred modes of education were: ‘books and printed information for self-study’ (45%), ‘web-based information for self-study’ (70%) and ‘online workshops [mixture of presentations and group activities]’ (70%) (Supplementary Material).

#### 3.1.2 Confidence

Prior to workshop participation, self-confidence in knowledge of genetic-related practice was low, with at least 50% of participants indicating that they were either ‘not at all confident’ or ‘a little confident’ for all 25 domains. Over 60% of participants indicated that they were ‘not at all confident’ in 11 domains. The highest of which were ‘perform a pedigree analysis’ (87%), ‘interpret genetic testing results’ (83%), and ‘provide genetic education and mentoring to your peers’ (77%).

#### 3.1.3 Learning needs

Regarding education experience and learning needs, most participants indicated that they ‘have not learnt about but want to’, across all topics (range: 59%–76%). This was highest for: ‘interpretation of genetic testing’ (76%), ‘provision of supportive care related to genetic testing’ (75%), ‘facilitation of genetic testing’ (74%) and ‘patient education and communication related to genetics’ (74%).

### 3.2 Workshop survey responses

#### 3.2.1 Demographics

One hundred and six nurses and midwives participated in the three workshops. Participants were predominantly female (90%) and aged above 40 years old (70%) (Supplementary Material). Participants’ demographics were similar in terms of years of practice and years in current specialty. There was a notable difference in locality, with nearly 90% of in-person workshop participants working in metropolitan health services, compared to those who participated online (50%).

#### 3.2.2 Confidence

**FIGURE 3:**
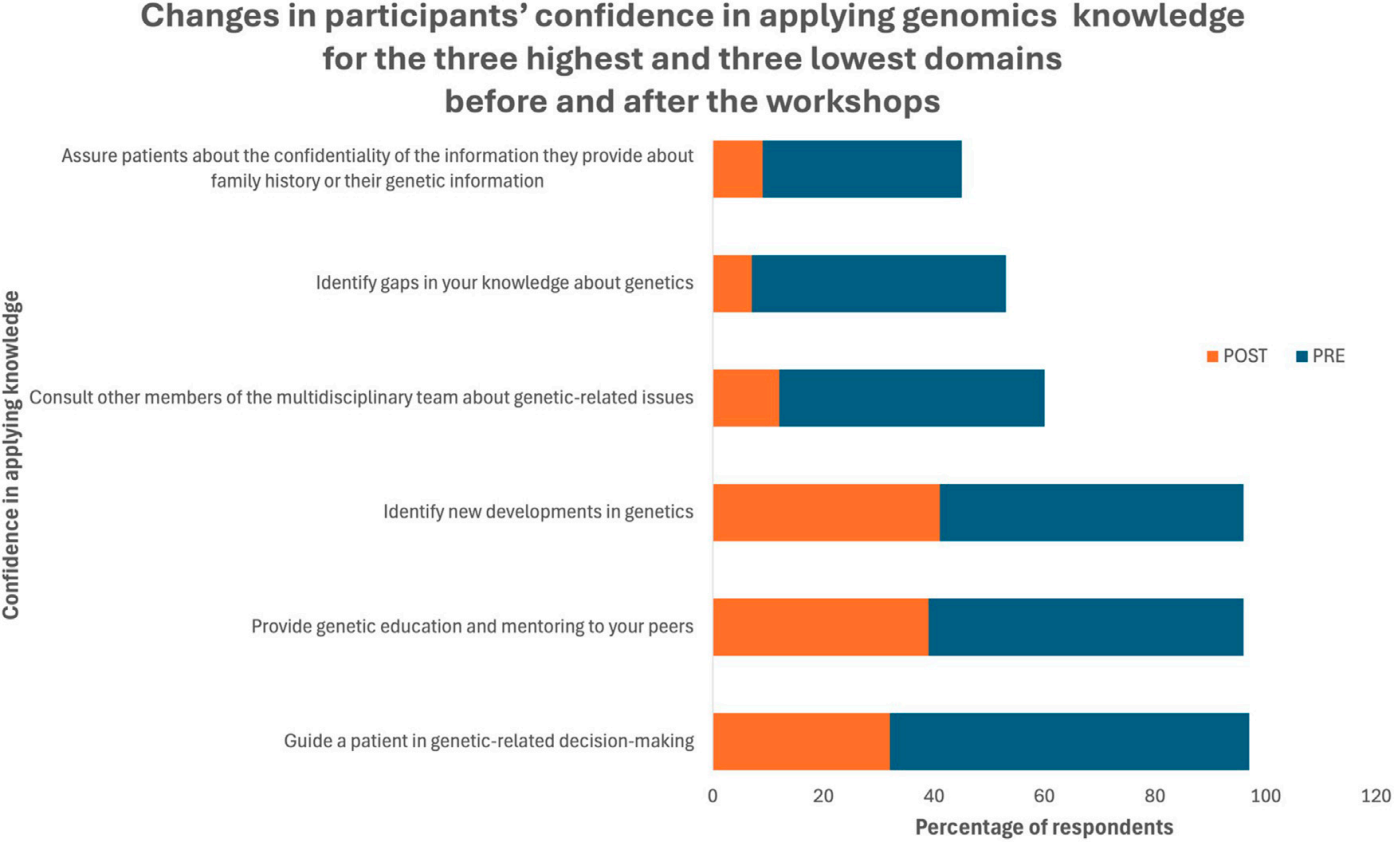
Confidence in Applying Genomics Knowledge.

The pre and post testing showed a change in confidence from ‘not at all confident’ to some level of confidence that was statistically significant (*P* < 0.001) across all 25 topics (Figure 3 and Supplementary Material). The biggest improvement in confidence was to ‘perform a pedigree analysis’, this changed from 76% or participants feeling ‘not at all confident’ before the workshops to just 4% after the workshops.

The smallest difference in participants indicating that they were ‘not at all confident’ was in response to ‘assure patients about confidentiality’ which went from 24% to 3% (two participants).

#### 3.2.3 Learning needs

**FIGURE 4:**
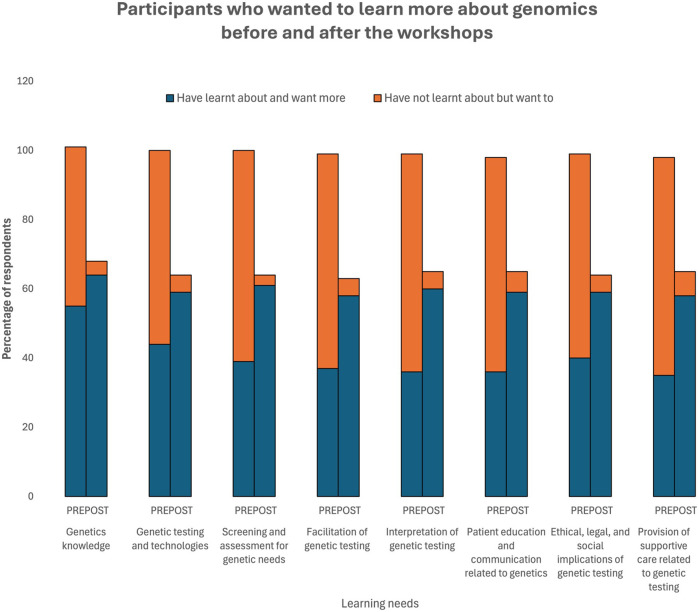
Self-Identified Learning Needs.

Participants wanted more education across all eight educational topics covered in the workshops (Figure 4 and Supplementary Material). The change in learning needs was statistically significant for all topics except for “genetics knowledge”. Prior to the workshop 52% of participants stated, “they have learnt about and want more”, this increased to 89% following the workshop.

The largest change in learning needs was for “interpretation of genetic testing”. Prior to the workshop 34% of participants indicated that “they have learnt about and want more” this increased to 86% following the workshop.

### 3.3 Workshop feedback

#### 3.3.1 Workshop one

Eleven of 21 participants completed feedback and evaluation forms. Of these, five participants (45%) indicated concern over the content being too advanced, with three participants suggesting pre-reading would be a way to address this. Eight (73%) mentioned a lack of time or going too fast, for example:

Too much emphasis on group responses… Maybe 2–3 days [instead of a 1-day workshop]. Too much info not discussed for lack of time.

Three (27%) mentioned issues with the online quiz, two being technical issues relating to the link and format, and one that the quiz was more difficult than expected.

Positives feedback included comments around the opportunity to work in small groups with people from similar areas and the networking opportunities (n = 5, 45%), the presenters’ expertise (n = 2, 18%), the consent section (n = 2, 18%), and the workbook (n = 2, 18%). Comments were also received about one activity where participants were asked to draw a pedigree for a hypothetical patient (n = 4, 36%). Two people would have liked more time for the activity, and one person felt that the activity might be more effective if undertaken on the screen in front of the class.

As a result of this feedback, pre-reading was introduced prior to workshops; the pedigree activity was undertaken on the screen, with audience participation; a QR code was generated which linked to the online quiz and presenters did not ask each group to present their responses after every activity.

#### 3.3.2 Workshop two

Of the 30 participants who provided feedback, only three (10%) felt that the pitch (level) of the workshop was problematic, and this feedback was generally worded more gently, for example:

[start] assessing knowledge before workshops to tailor learning.

*Some of it was way over our heads but still very interesting*.

Compared with feedback from Workshop One:

It has been good to have some background knowledge. For nurses/midwives who do not have that then this whole day would be overwhelming.

There was only one mention of time constraints, and again much gentler language around was used:

Possibly start early so not in a rush during lecture.

For Workshop Two there was one positive comment about the quiz, and no negative comments, compared with three (27%) negative comments after Workshop One.

There were three positive comments on the pre-reading given. These were asking for more or more specific readings, and for more time to read it, one stating that participants should read the whole workbook prior to the workshop. However, one negative comment about the pre-reading stated they believed the content in the workshop could be reduced to avoid repetition of the pre-reading content.

For the ‘keep doing’ prompt, 16 participants (53%) gave a response of ‘what you’re doing!’ or similar. Five (17%) articulated a positive experience with the presenters, for example:

Nice to have presenters so passionate in your field of practice! Very well presented, all modules well organised.

There were four positive comments around the workbook, for example:

Education resource is excellent-well set out and written well.

The booklet is great to take home and allowed for more listening during sessions, and review.

However, six (20%) participants referenced an issue with following the order of the presentation from the workbook, an issue which was not mentioned in the first workshop.

Seven participants (23%) commented how they will use their new knowledge in practice and sharing with their colleagues, for example:

Will share what I’ve learnt with my colleagues.

Definitely encouraged me to develop my interest further in this area.

There were five comments on the positive experience of the group work, for example:

[keep doing] group activities … mix of background in genomic/nursing areas.

Fifteen (50%) participants expressed gratitude for the course, for example:

Thoroughly enjoyed the session. It has been beneficial to me and has demonstrated the important role of a nurse in genomics.

Based on the feedback, the following changes were made: participants received a copy of the slides ahead of workshop three and a glossary was added to the workbook. These changes are in line with the universal Design for Learning Guidelines which can be used to meet the needs of all learners (CAST, 2018).

#### 3.3.3 Workshop three

Of the 38 participants who attended the third, online, workshop, 21 participants gave feedback. There was very little feedback in the ‘stop doing’ category (n = 4, 19%). One participant commented on difficulty using their device for the quiz, one commented that the first workshop was complex but acknowledged this was due to novelty and not something that could likely be amended. Two participants commented negatively on the group work; one found the activities challenging due to a lack of familiarity with the application of the concepts being discussed, and one requested less group work. This was however balanced by six positive comments about the group work, outlining the benefits of cross-specialty networking, for example:

Have enjoyed the online sessions - in particular seeing and hearing the different specialties and geographic areas we all practice in. Helps to change your perspective of what is common for you, may not be for someone else. Break out rooms a good idea to provide more small group time.

There were four comments in the ‘start doing’ category, three outlining a desire for further education in genomics and one asked to see a particular concept demonstrated.

Sixteen (76%) participants gave feedback in the ‘keep doing’ section. There was specific feedback about the positives of online learning from eight participants, such as:

I liked [that] the session was delivered in bite size chunks. I imagine a full day workshop would be very full on and some of this information, language *etc.*, is quite difficult. Having time between each session allowed time to reflect and being fresh to take on more information.

I did not think I would prefer the online learning as much but actually found it to be really well organised.

In contrast to feedback from the first workshop, the complexity of the content was only mentioned once, as a positive aspect:

I found the course very informative. The information was pitched at a good level.

Five participants (24%) commented on the practical application of the course to their work, for example:

The course confirmed to me it is an area I am extremely interested in and gave me the confidence to pursue further.

It has inspired me to improve my knowledge in this area. To explore ways to integrate it into my current practice. To consider becoming a genetic counsellor in the future.

[The course] has got me thinking about being more proactive in my role in genetic referral and digging a bit deeper with clients about their motivation (or lack of) for testing.

The were also positive comments about the presenters, for example:

The presenters and content was [sic] excellent and engaging, my interest in further learning was encouraged and my questions during the presentations were answered. Thanks for an excellent learning experience.

The delivery of the workshop in three sessions was received positively, as it allowed participants time to ‘digest’ information from each session. Going forward ways to continue this staged delivery both in-person and through online sessions will be explored, e.g., lunchtime workshops. Further an e-newsletter could be developed and circulated to keep attendees abreast of developments in the area, including both conferences and courses.

## 4 Discussion

**FIGURE 5:**
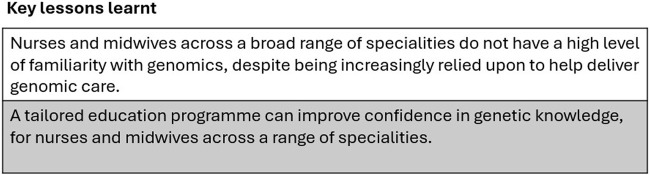
Key Lessons Learnt.

This project was developed to enhance the genomics capabilities of nurses and midwives. Key lessons learnt from this project are highlighted in Figure 5. The knowledge domains identified through the formative research were similar to those found in qualitative interviews that identified the perceived genetic knowledge and education needs of Australian allied health professionals, nurses and midwives (Saleh et al., 2019). A lack of knowledge of genetics in nursing staff is a significant barrier to mainstreaming genomics in healthcare (White et al., 2020). Our results indicate that there is a strong desire amongst nurses and midwives to upskill their genetic literacy. Similar findings have been found with specialists both in Australia and overseas (Crellin et al., 2019; McClaren et al., 2020a). Participants appreciated cross-specialty involvement, which allowed them to draw similarities from other nursing areas into their own practice. Similar to our findings, Saleh reported that genomics is rarely covered in undergraduate degrees or in formal genetics training, although on-the-job training may fill some of these gaps (Saleh et al., 2019). This poses a significant challenge in screening and assessing people for their genomic needs in order to facilitate optimum care (Barr et al., 2018) and underscores the importance of our work. Following their review Saleh et al. (2019) called for an education programme.

Prior to the genomics education workshop, confidence in applying genomics knowledge and skills was poor, with most participants stating they were either ‘not at all confident’, or only ‘a little confident’ across a broad range of current practice areas (Figure 4). This finding echoes those from Australian general practitioners and health interpreters, who also reported lack of confidence in this area (Cusack et al., 2021; Vidgen et al., 2021).

One of the important aspects of developing the genomics education programme was to ensure that nursing and midwifery professionals’ perspectives were embedded in each iteration of the design. The continuous workshop improvements based on evaluation data facilitated the creation of an education programme that was designed specifically to meet the educational needs of our cohort. Understanding the needs of our target audience to inform the development of the workshop was vital to our educational design and has been recognised by other researchers (McClaren et al., 2020b). In the future, it would also be useful to consider the perspectives of others when developing such an education programme, including other healthcare professionals, the health consumer and their family. Each of these groups bring their own unique perspective on the most important elements of genomic education (Whitley et al., 2020; Cusack et al., 2021).

Although the perspective of the health consumer was not explicitly sought, the authors noted that several workshop participants also had experience with genomics as a health consumer. These were identified mostly through one-on-one conversations with the presenters between the formal sessions. One participant brought a letter she received from her genomics team 10 years prior and presented it as evidence of how poorly the system handled her needs. This lived experience motivated these nurses and midwives to work in areas with genetic conditions and to be educated and provide better care to their own patients. Personal experience was also seen to engage healthcare interpreters in basic genetics training (Vidgen et al., 2021).

Confidence in all domains improved following the workshops, which reflects the findings from a similar study, with nurses who possessed a doctoral degree or were doctoral students (Kronk et al., 2024). We identified a persistent level of under confidence with interpreting genetic testing results and educating patients and their families about genetic susceptibility (Figure 3). These two competencies were included based on the UK and US nursing competencies, where genetic counselling is part of a registered nurses role (Nursing and Midwifery Council, 2018; American Association of Colleges of Nursing, 2020). In Australia, these activities more typically lie within the remit of qualified genetic counsellors. However, both the Human Genetics Society of Australasia and the Australian Genomics Health Alliance have reported that the Australian genetic counselling workforce is less than half that recommended by international benchmarking and is currently not meeting the needs of the population (Nisselle et al., 2019a; Allied Health Professions Australia, 2022). This limited number of genetic counsellors, paired with the mainstreaming of genomics throughout healthcare, means that nurses and midwives are now required to interpret and communicate genetic testing results.

The biggest improvements in confidence were seen in areas that were probably not covered by the nursing curriculum at the time of training nor widely practiced, such as performing a pedigree analysis. Conversely, topics that saw smaller increases in confidence are those well covered in the nursing curriculum and regularly performed, such as ‘assuring patients about confidentiality’.

It is apparent that proper genomic literacy for nursing staff is critical for contemporary medical care and that this can be improved through continuing education. This becomes imperative as nurses are more frequently required to facilitate patient referral or provide patient education following a diagnosis for the patient.

Interpreting genetic test results is challenging, even for genetics professionals (Donohue et al., 2021). Concerns around interpreting such results have been raised elsewhere, and upskilling in the ability to contextualise the information provided is also needed (Flowers et al., 2020). While it seems that medical geneticists and genetic counsellors are the least likely to misinterpret test results, nurses seem to be adequate compared to their general practitioner and specialist colleagues (Donohue et al., 2021). As nurses are the most trusted profession in Australia (Morgan, 2021), patients often open up to them more often than their doctor–which provides the need for nursing staff to be able to confidently answer questions from their patients (Dinc and Gastmans, 2013).

Some strengths of this study were the formative research which identified the learning preferences and genomics training needs of the target cohort, as well as the inclusion of nurses and midwives working in both metropolitan and rural/regional areas across Queensland. However, the results may not reflect the broader educational needs of nurses and midwives across all of Australia. A further strength of this work is that it helps increase understanding of genomics by nurses and midwives outside of the United States of America (Thomas et al., 2023).

While feedback from workshop three (online) was overwhelmingly positive, it must be noted that this option was the only one which allowed for attrition. From 107 registered individuals, 58 participants attended the first workshop, 40 attended the second, and 38 the third. Therefore, it is plausible that those who did not like the format did not return to subsequent workshops. Another limitation of this research is that we only assessed confidence. Despite its theoretical basis, it is unknown whether increased confidence is reflected in practice changes. While we used the SQUIRE-EDU reporting tool (Ogrinc et al., 2019), the authors suggest that future work is reported in line with RISE2 Genomics (Nisselle et al., 2019b).

A further limitation of this research was the use of a convenience sample of nurses and midwives who were motivated to learn more about genomics. Future research should aim to explore the perspectives of other healthcare practitioners, health consumers, and their families. It should also investigate how increased confidence in genomics impacts the practice of nurses and midwives.

### 4.1 Conclusion

Nurses and midwives are not confident in their knowledge of genetics, despite being increasingly relied upon to help deliver genetic care. We showed that this lack of confidence can be successfully addressed using a tailored education programme specifically designed to upskill nurses and midwives in areas of genetics relevant to their scope of practice, regardless of their area of speciality practice.

## Data Availability

The original contributions presented in the study are included in the article/Supplementary Material, further inquiries can be directed to the corresponding author.
