# Supplementary material for: Assessing genomics confidence and learning needs in Australian nurses and midwives: an educational program evaluation
Source: Front Genet. 2024 Jul 16;15:1419302. doi: 10.3389/fgene.2024.1419302 (PMC11286479; doi:10.3389/fgene.2024.1419302)
Supplement: Supplementary file 1 [file Table1.DOCX]

Supplementary Material

# Supplementary Data

**Table 1:** **Genetics Education Survey for Queensland Nurses and Midwives**

| GENETICS PRACTICE CONFIDENCE | | |
| --- | --- | --- |
| Thinking about your current practice and the area in which you work, how confident are you that you can: | | Answer options:  1 Not at all confident  2 A little confident  3 Confident  4 More confident  5 Very confident  6 Not applicable |
| Topic: Genetics knowledge | |  |
|  | Understand relevant genetic information |  |
| Topic: Genetic testing and technologies | |  |
|  | Understand relevant genetic tests and technologies |  |
| Topic: Screening and assessment for genetic needs | |  |
|  | Identify clinical indicators of genetic susceptibility |  |
|  | Take a family history |  |
|  | Perform a pedigree analysis |  |
| Topic: Facilitation of genetic testing | |  |
|  | Decide which patients would benefit from a referral for genetic services |  |
|  | Facilitate referral for genetic services |  |
|  | Contact genetic services for a consultation for a patient |  |
|  | Consult with a nursing or midwifery peer about a genetic-related issue |  |
|  | Consult other members of the multidisciplinary team about genetic-related issues |  |
|  | Advocate for access to desired genetic services |  |
| Topic: Interpretation of genetic testing | |  |
|  | Interpret genetic testing results |  |
| Topic: Patient education and communication related to genetics | |  |
|  | Provide information about genetic testing |  |
|  | Educate a patient or their family about genetic susceptibility |  |
|  | Answer questions about genetics by a patient |  |
| Topic: Provision of supportive care related to genetic testing | |  |
|  | Respond appropriately to a patient experiencing distress related to genetic issues |  |
|  | Incorporate genetic information into care provided |  |
| Topic: Ethical, legal and social implications of genetic testing | |  |
|  | Guide a patient in genetic-related decision-making |  |
|  | Inform patients about the risks, benefits and limitations of genetic testing |  |
|  | Assure patients about the confidentiality of the information they provide about family history or their genetic information |  |
|  | Recognise the ethical issues associated with taking a family history or genetic testing |  |
| Topic: Professional development | |  |
|  | Provide genetic education and mentoring to your peers |  |
|  | Identify new developments in genetics |  |
|  | Identify gaps in your knowledge about genetics |  |
|  | Participate in the conduct of genetic-related research |  |
|  | Participate in genetic-related professional development activities |  |
|  | Access current and reliable information |  |
| GENETICS EDUCATION NEEDS | | |
| Please indicate your education experience and learning needs in the following topics: | | Answer options:  1 Have learnt about and want more  2 Have learnt about and do not want more  3 Have not learnt about but want to  4 Have not learnt about and do not want to  5 Unsure  6 Not applicable |
| Genetics knowledge | |  |
| Genetic testing and technologies | |  |
| Screening and assessment for genetic needs | |  |
| Facilitation of genetic testing | |  |
| Interpretation of genetic testing | |  |
| Patient education and communication related to genetics | |  |
| Provision of supportive care related to genetic testing | |  |
| Ethical, legal and social implications of genetic testing | |  |
| Engaging in professional development opportunities related to genetics | |  |
| GENETICS EDUCATION PREFERENCES | | |
| How helpful would you find each of these formats for learning about genetics? | | Answer Options  1 Not at all helpful  2 A little helpful  3 Helpful  4 Some-what helpful  5 Most helpful |
| Books and printed information for self-study | |  |
| Web based information for self-study | |  |
| A mix of both of the above | |  |
| A mix of self-study and face-to-face learning | |  |
| Face-to-Face Workshops [mixture of presentations and group activities] | |  |
| Face-to-Face Presentations | |  |
| Online Workshops [mixture of presentations and group activities] | |  |
| Online Presentations | |  |
| A full day program of genetic education | |  |
| A series of smaller genetic education modules/workshops/presentations | |  |

**Table 2: Demographic information for participants**

|  | | **Pre-development of Workshops Survey** | **Workshop 1 and 2  (In-Person)** | | **Workshop 3  (Online)** | | **All Participants** | |
| --- | --- | --- | --- | --- | --- | --- | --- | --- |
|  | |  | **Pre-workshop** | **Post-workshop** | **Pre-workshop** | **Post-workshop** | **Pre-workshop** | **Post-workshop** |
| **Survey Question** | **Answer** | **n=274** | **n=59** | **n=50** | **n=47** | **n=24*** | **n=106** | **n=74** |
| Age (years) | 18-29  30-39  40-49  50-59  60+ | 18(6.6)  64(23.4)  83(30.3)  95(34.7)  14(5.0) | 5(8.5)  13(22.0)  17(28.8)  20(33.9)  4(6.8) | 4(8.0)  12(24.0)  14(28.0)  17(34.0)  3(6.0) | 2(4.3)  12(25.5)  13(27.7)  16(34.0)  4(8.5) | 0  6(25.0)  9(37.5)  6(25.0)  3(12.5) | 7(6.6)  25(23.6)  30(28.3)  36(34.0)  8(7.5) | 4(5.4)  18(24.3)  23(31.1)  23(31.1)  6(8.1) |
| Gender | Female  Male  Other | 243(89.3)  29(10.7)  0 | 51(86.4)  8(13.6)  0 | 47(86.0)  7(14.0)  0 | 44(93.6)  3(6.4)  0 | 21(87.5)  3(12.5)  0 | 95(89.6)  11(10.4)  0 | 64(86.5)  10(13.5)  0 |
| Location | Metropolitan  Regional  Rural/Remote | 179(65.3)  63(23.0)  32(11.7) | 53(89.8)  6(10.2)  0 | 45(90.0)  5(10.0)  0 | 24(51.1)  19(40.4)  4(8.5) | 11(45.9)  11(45.8)  2(8.3) | 77(72.6)  25(23.6)  4(3.8) | 56(75.7)  16(21.6)  2(2.7) |
| Years of practice experience | < 10 years  10-20 years  > 20 years | 54(20.1)  69(25.8)  145(54.1) | 11(18.6)  17(28.9)  31(52.5) | 9(18.8)  15(31.2)  24(50.0) | 7(14.9)  17(36.2)  23(48.9) | 1(4.3)  9(39.1)  13(56.6) | 18(17.0)  34(32.1)  54(50.9) | 10(14.1)  24(33.8)  37(52.1) |
| Years of practice experience in the area you work in now | < 10 years  10-20 years  > 20 years | 120(44.1)  89(32.7)  63(23.2) | 25(43.1)  19(32.8)  14(24.1) | 22(44.0)  14(28.0)  14(28.0) | 20(42.6)  20(42.6)  7(14.8) | 9(37.5)  8(33.3)  7(29.2) | 45(42.9)  39(37.1)  21(20.0) | 31(41.9)  22(29.7)  21(28.4) |
| Registration | Registered Nurse  Registered Midwife  Both | 226(82.8)  8(2.9)  39(14.3) | 49(83.0)  3(5.1)  7(11.9) | 42(84.0)  2(4.0)  6(12.0) | 34(77.2)  1(2.3)  9(20.5) | 18(75.0)  0  6(25.0) | 83(80.6)  4(3.9)  16(15.5) | 60(81.1)  2(2.7)  12(16.2) |
| Main role | Registered Nurse  Registered Midwife  Clinical Nurse/Midwife  Unit Manager  Manager  Educator  Researcher  Nurse Navigator  Nurse Practitioner  Other | 65(23.8)  7(2.6)  85(31.0)  19(6.9)  11(4.0)  28(10.2)  6(2.2)  13(4.7)  8(2.9)  32(11.7) | 17(28.8)  2(3.4)  15(25.4)  3(5.1)  3(5.1)  4(6.8)  2(3.4)  2(3.4)  4(6.8)  7(11.8) | 16(32.0)  2(4.0)  13(26.0)  3(6.0)  2(4.0)  3(6.0)  2(4.0)  2(4.0)  2(4.0)  5(10.0) | 12(26.1)  1(2.2)  19(41.3)  3(6.5)  1(2.2)  2(4.3)  1(2.2)  0  0  7(15.2) | 1(4.2)  1(4.2)  14(58.3)  1(4.2)  0(0.0)  2(8.3)  0(0.0)  2(8.3)  0  3(12.5) | 29(27.6)  3(2.9)  34(32.4)  6(5.7)  4(3.8)  6(5.7)  3(2.9)  2(1.9)  4(3.8)  14(13.3) | 17(23.0)  3(4.1)  27(36.5)  4(5.4)  2(2.7)  5(6.7)  2(2.7)  4(5.4)  2(2.7)  8(10.8) |
| Highest Qualification | Hospital Certificate  Post Registration Certificate  Diploma  Bachelor Degree  Postgraduate | 7(2.6)  23(8.5)  12(4.4)  76(27.9)  154(56.6) | 3(5.1)  5(8.4)  3(5.1)  19(32.2)  29(49.2) | 1(2.0)  5(10.0)  3(6.0)  19(38.0)  22(44.0) | 1(2.1)  2(4.3)  3(6.4)  7(14.9)  34(72.3) | 1(4.2)  2(8.3)  2(8.3)  6(25.0)  13(54.2) | 4(3.8)  7(6.6)  6(5.7)  26(24.5)  63(59.4) | 2(2.7)  7(9.5)  5(6.8)  25(33.7)  35(47.3) |
| Postgraduate | Graduate Certificate/Diploma  Masters (Coursework)  Masters (Research)  PhD | 83(53.9)  61(39.6)  6(3.9)  4(2.6) | 14(48.3)  13(44.9)  1(3.4)  1(3.4) | 11(50.0)  9(41.0)  1(4.5)  1(4.5) | 20(58.8)  10(29.4)  4(11.8)  0 | 8(61.5)  4(30.8)  1(7.7)  0 | 34(54.0)  23(36.5)  5(7.9)  1(1.6) | 19(54.3)  13(37.1)  2(5.7)  1(2.9) |
| Type of Employment | Full-time  Part-time  Casual | 145(53.3)  123(45.2)  4(1.5) | 27(45.8)  29(49.2)  3(5.0) | 22(44.0)  25(50.0)  3(6.0) | 24(51.0)  21(44.7)  2(4.3) | 10(43.5)  13(56.5)  0 | 51(48.1)  50(47.2)  5(4.7) | 32(43.8)  38(52.1)  3(4.1) |
| Main Specialty/ Practice Area | Cancer  Cardiothoracic  Community Health  Emergency  Endocrine  Infection Control  Informatics  Intensive Care  Maternity/Neonatal  Mental Health  Neurology  Paediatric  Renal  Research  Rural Health  Surgical/Perioperative  Other (Medical) | 25(9.7)  18(7.0)  29(11.3)  11(4.3)  6(2.2)  6(2.3)  4(1.6)  8(3.1)  28(10.9)  14(5.4)  3(1.2)  10(3.9)  8(3.1)  21(8.2)  4(1.6)  30(11.7)  32 (12.5) | 11(18.5)  2(3.4)  2(3.4)  2(3.4)  3(5.1)  1(1.6)  0(0.0)  2(3.4)  9(15.3)  0  3(5.1)  9(15.3)  0  3(5.1)  0  3(5.1)  9(15.3) | 9(18.4)  2(4.1)  1(2.0)  1(2.0)  2(4.1)  1(2.0)  0  2(4.1)  7(14.3)  0  2(4.1)  7(14.3)  0  3(6.1)  0  3(6.1)  9(18.4) | 9(25.7)  3(8.6)  0  0  1(2.8)  3(8.6)  0  2(5.7)  4(11.4)  0  1(2.9)  4(11.4)  0  2(5.7)  0  1(2.9)  5(14.3) | 9(42.8)  3(14.3)  0  0  0  2(9.5)  0  0  2(9.5)  0  1(4.8)  2(9.5)  0  1(4.8)  0  0  1(4.8) | 20(18.5)  4(3.7)  3(2.8)  3(2.8)  5(4.6)  2(1.8)  0  4(3.7)  16(14.8)  0  5(4.6)  16(14.8)  0  6(5.6)  0  6(5.6)  18(16.7) | 18(32.1)  6(10.7)  0  0  1(1.8)  5(8.9)  0  2(3.6)  6(10.7)  0  2(3.6)  6(10.7)  0  3(5.4)  0  1(1.8)  6(10.7) |

**Notes:** *Three of the 24 participants (12.5%) missed one of four online workshops.

**Table 3: Genomics education preferences used to inform the development of workshops**

| **n = 261** | | |
| --- | --- | --- |
| **Survey Question** | **Answer** | **n (%)** |
| **How helpful would you find each of these formats for learning about genetics?** |  |  |
| Books and printed information for self-study | Not helpful  Somewhat helpful  Unsure  Quite helpful  Very helpful | 19(7.4)  102(39.5)  20(7.8)  95(36.8)  22(8.5) |
| Web based information for self-study | Not helpful  Somewhat helpful  Unsure  Quite helpful  Very helpful | 9(3.5)  55(21.4)  14(5.4)  142(55.3)  37(14.4) |
| A mix of books, printed, and web-based information for self-study | Not helpful  Somewhat helpful  Unsure  Quite helpful  Very helpful | 12(4.6)  46(17.8)  22(8.5)  119(45.9)  60(23.2) |
| A mix of self-study and face-to-face learning | Not helpful  Somewhat helpful  Unsure  Quite helpful  Very helpful | 9(3.5)  25(9.7)  16(6.2)  118(45.7)  90(34.9) |
| Face-to-Face Workshops [mixture of presentations and group activities] | Not helpful  Somewhat helpful  Unsure  Quite helpful  Very helpful | 11(4.2)  22(8.5)  19(7.3)  105(40.4)  103(39.6) |
| Face-to-Face Presentations | Not helpful  Somewhat helpful  Unsure  Quite helpful  Very helpful | 6(2.3)  26(10.1)  19(7.4)  100(39.0)  106(41.2) |
| Online Workshops [mixture of presentations and group activities] | Not helpful  Somewhat helpful  Unsure  Quite helpful  Very helpful | 12(4.6)  35(13.5)  31(11.9)  120(46.2)  62(23.8) |
| Online Presentations | Not helpful  Somewhat helpful  Unsure  Quite helpful  Very helpful | 5(1.9)  32(12.3)  23(8.8)  134(51.3)  67(25.7) |
| A full day programme of genetic education | Not helpful  Somewhat helpful  Unsure  Quite helpful  Very helpful | 13(5.0)  29(11.2)  34(13.1)  98(37.9)  85(32.8) |
| A series of smaller genetic education modules/workshops/presentations | Not helpful  Somewhat helpful  Unsure  Quite helpful  Very helpful | 5(2.0)  21(8.0)  25(9.6)  111(42.5)  99(37.9) |

**Table 4: Confidence in applying knowledge of genomics and related procedures to practice**

| **Survey Question** | **Answer** | **Pre-workshop Development** | **Workshop 1 and 2**  **(In-Person)** | | **Workshop 3 (Online)** | | **All Workshop Participants** | | ***p* value*** |
| --- | --- | --- | --- | --- | --- | --- | --- | --- | --- |
|  |  |  | **Pre** | **Post** | **Pre** | **Post** | **Pre** | **Post** |  |
| **Thinking about your current practice and the area in which you work, how confident are you that you can:** | | n=270 | n=59 | n=50 | n=46 | n=22 | n=105 | n=74 |  |
| Identify clinical indicators of genetic susceptibility | Not at all confident  A little confident  Confident  More confident  Very Confident | 133(51.0)  92(35.2)  21(8.0)  6(2.3)  9(3.5) | 24(40.7)  21(35.6)  8(13.6)  5(8.4)  1(1.7) | 4(8.2)  17(34.7)  5(10.2)  20(40.8)  3(6.1) | 25(54.4)  16(34.4)  4(8.9)  0(0.0)  1(2.3) | 1(4.5)  7(31.9)  3(13.6)  10(45.5)  1(4.5) | 49(46.7)  37(35.2)  12(11.4)  5(4.8)  2(1.9) | 5(7.0)  24(33.8)  8(11.3)  30(42.3)  4(5.6) | <0.001 |
| Perform a pedigree analysis | Not at all confident  A little confident  Confident  More confident  Very Confident | 222(86.7)  20(7.8)  5(2.0)  2(0.8)  7(2.7) | 42(71.2)  8(13.6)  3(5.1)  1(1.6)  5(8.5) | 3(6.1)  22(44.9)  9(18.4)  12(24.5)  3(6.1) | 38(82.6)  4(8.7)  2(4.3)  1(2.2)  1(2.2) | 0  12(52.2)  4(17.4)  4(17.4)  3(13.0) | 80(76.2)  12 (11.4)  5(4.8)  2(1.9)  6(5.7) | 3(4.2)  34(47.2)  13(18.1)  16(22.2)  6(8.3) | <0.001 |
| Decide which patients would benefit from a referral for genetic services | Not at all confident  A little confident  Confident  More confident  Very Confident | 147(57.4)  78(30.5)  20(7.8)  7(2.7)  4(1.6) | 21(36.2)  21(36.2)  11(19.0)  4(6.9)  1(1.7) | 4(8.3)  17(35.4)  11(22.9)  14(29.2)  2(4.2) | 25(54.3)  16(34.8)  5(10.9)  0  0 | 0  5(21.8)  6(26.1)  11(47.8)  1(4.3) | 46(44.2)  37(35.6)  16(15.4)  4(3.8)  1(1.0) | 4(5.6)  22(31.0)  17(24.0)  25(35.2)  3(4.2) | <0.001 |
| Facilitate referral for genetic services | Not at all confident  A little confident  Confident  More confident  Very Confident | 170(65.9)  49(19.0)  23(8.9)  10(3.9)  6(2.3) | 31(54.4)  13(22.8)  9(15.8)  2(3.5)  2(3.5) | 5(10.6)  12(25.5)  11(23.4)  13(27.7)  6(12.8) | 31(67.4)  8(17.4)  4(8.7)  1(2.2)  2(4.3) | 0  3(13.6)  8(36.4)  3(13.6)  8(36.4) | 62(60.2)  21(20.4)  13(12.6)  3(2.9)  4(3.9) | 5(7.2)  15(21.8)  19(27.5)  16(23.2)  14(20.3) | <0.001 |
| Contact genetic services for a consultation for a patient | Not at all confident  A little confident  Confident  More confident  Very Confident | 153(60.2)  54(21.3)  28(11.0)  10(4.0)  9(3.5) | 30(54.5)  10(18.2)  10(18.2)  2(3.6)  3(5.5) | 3(6.4)  15(31.9)  13(27.7)  9(19.1)  7(14.9) | 28(60.9)  11(23.9)  6(13.0)  1(2.2)  0 | 0  3(13.6)  8(36.4)  3(13.6)  8(36.4) | 58(57.4)  21(20.8)  16(15.8)  3(3.0)  3(3.0) | 5(4.3)  18(26.1)  21(30.4)  12(17.4)  15(21.8) | <0.001 |
| Consult with a nursing or midwifery peer about a genetic-related issue | Not at all confident  A little confident  Confident  More confident  Very Confident | 101(37.8)  81(30.2)  58(21.6)  10(3.7)  18(6.7) | 16(27.6)  15(25.8)  23(39.7)  1(1.7)  3(5.2) | 0  8(17.0)  17(36.2)  10(21.3)  12(25.5) | 18(39.1)  20(43.5)  6(13.0)  1(2.2)  1(2.2) | 0  3(12.5)  8(33.3)  9(37.5)  4(16.7) | 34(32.7)  35(33.7)  29(27.9)  2(1.9)  4(3.8) | 0  11(15.5)  25(35.2)  19(26.8)  16(22.5) | <0.001 |
| Consult other members of the multidisciplinary team about genetic-related issues | Not at all confident  A little confident  Confident  More confident  Very Confident | 92(34.6)  78(29.3)  59(22.2)  15(5.6)  22(8.3) | 15(25.4)  14(23.7)  20(33.9)  6(10.2)  4(6.8) | 0  10(20.7)  14(29.2)  15(31.3)  9(18.8) | 16(34.8)  15(32.6)  11(23.9)  3(6.5)  1(2.2) | 0  2(8.8)  7(30.4)  7(30.4)  7(30.4) | 31(29.5)  29(27.6)  31(29.5)  9(8.6)  5(4.8) | 0  12(16.9)  21(29.6)  22(31.0)  16(22.5) | <0.001 |
| Inform patients about the risks, benefits and limitations of genetic testing | Not at all confident  A little confident  Confident  More confident  Very Confident | 180(68.7)  54(20.6)  19(7.3)  6(2.3)  3(1.1) | 33(55.9)  16(27.1)  9(15.3)  0  1(1.7) | 2(4.2)  19(39.6)  10(20.7)  14(29.2)  3(6.3) | 28(60.8)  13(28.3)  4(8.7)  0  1(2.2) | 0  5(21.8)  10(43.5)  7(30.4)  1(4.3) | 61(58.1)  29(27.6)  13(12.4)  0  2(1.9) | 2(2.8)  24(33.8)  20(28.2)  21(29.6)  4(5.6) | <0.001 |
| Interpret genetic testing results | Not at all confident  A little confident  Confident  More confident  Very Confident | 220(83.3)  30(11.4)  7(2.7)  3(1.1)  4(1.5) | 41(70.7)  11(19.0)  4(6.9)  1(1.7)  1(1.7) | 18(37.5)  20(41.6)  3(6.3)  6(12.5)  1(2.1) | 34(73.9)  8(17.4)  3(6.5)  1(2.2)  0 | 3(12.5)  13(54.2)  6(25.0)  2(8.3)  0 | 75(72.1)  19(18.3)  7(6.7)  2(1.9)  1(1.0) | 21(29.2)  33(45.8)  9(12.5)  8(11.1)  1(1.4) | <0.001 |
| Provide information about genetic testing | Not at all confident  A little confident  Confident  More confident  Very Confident | 185(69.5)  58(21.8)  12(4.5)  6(2.3)  5(1.9) | 26(45.6)  23(40.4)  5(8.7)  2(3.5)  1(1.8) | 3(6.3)  20(41.6)  14(29.2)  9(18.7)  2(4.2) | 28(60.9)  14(30.4)  3(6.5)  1(2.2)  0 | 0  7(29.2)  11(45.8)  6(25.0)  0 | 54(52.4)  37(35.9)  8(7.8)  3(2.9),  1(1.0) | 3(4.2)  27(37.5)  25(34.7)  15(20.8)  2(2.8) | <0.001 |
| Educate a patient or their family about genetic susceptibility | Not at all confident  A little confident  Confident  More confident  Very Confident | 192(73.3)  52(19.8)  11(4.2)  5(1.9)  2(0.8) | 33(57.9)  17(29.8)  4(7.0)  2(3.5)  1(1.8) | 8(16.3)  20(40.9)  11(22.4)  8(16.3)  2(4.1) | 35(76.1)  7(15.2)  4(8.7)  0  0 | 1(4.3)  12(52.2)  5(21.7)  5(21.7)  0 | 68(66.0)  24(23.3)  8(7.8)  2(1.9)  1(1.0) | 9(12.5)  32(44.4)  16(22.2)  13(18.1)  2(2.8) | <0.001 |
| Respond appropriately to a patient experiencing distress related to genetic issues | Not at all confident  A little confident  Confident  More confident  Very Confident | 136(50.7)  80(29.8)  35(13.1)  12(4.5)  5(1.9) | 23(39.0)  29(49.2)  4(6.7)  2(3.4)  1(1.7) | 5(10.0)  10(20.0)  24(48.0)  8(16.0)  3(6.0) | 25(55.6)  12(26.6)  7(15.6)  1(2.2)  0 | 0  2(8.7)  12(52.2)  9(39.1)  0 | 48(46.2)  41(39.4)  11(10.6)  3(2.9)  1(0.9) | 5(6.9)  12(16.4)  36(49.3)  17(23.3)  3(4.1) | <0.001 |
| Guide a patient in genetic-related decision-making | Not at all confident  A little confident  Confident  More confident  Very Confident | 201(76.7)  43(16.4)  12(4.6)  1(0.4)  5(1.9) | 38(64.4)  14(23.7)  6(10.2)  0  1(1.7) | 7(14.9)  14(29.8)  15(31.9)  9(19.1)  2(4.3) | 35(77.8)  10(22.2)  0  0  0 | 0  11(47.9)  9(39.1)  3(13.0)  0 | 73(70.2)  24(23.1)  6(5.7)  0  1(1.0) | 7(10.0)  25(35.7)  24(34.3)  12(17.1)  2(2.9) | <0.001 |
| Answer questions about genetics by a patient | Not at all confident  A little confident  Confident  More confident  Very Confident | 178(66.4)  68(25.4)  15(5.6)  3(1.1)  4(1.5) | 29(49.2)  24(40.6)  5(8.5)  0  1(1.7) | 7(14.0)  25(50.0)  11(22.0)  5(10.0)  2(4.0) | 31(67.4)  11(23.9)  3(6.5)  1(2.2)  0 | 0  8(34.8)  10(43.5)  5(21.7)  0 | 60(57.1)  35(33.3)  8(7.6)  1(1.0)  1(1.0) | 7(9.6)  33(45.2)  21(28.8)  10(13.7)  2(2.7) | <0.001 |
| Access current and reliable genetic-related information | Not at all confident  A little confident  Confident  More confident  Very Confident | 153(56.7)  82(30.4)  22(8.1)  7(2.6)  6(2.2) | 19(32.8)  30(51.7)  8(13.8)  0  1(1.7) | 1(2.0)  16(32.0)  19(38.0)  9(18.0)  5(10.0) | 24(53.4)  14(31.1)  5(11.1)  1(2.2)  1(2.2) | 0  2(9.1)  11(50.0)  6(27.3)  3(13.6) | 43(41.8)  44(42.7)  13(12.6)  1(1.0)  2(1.9) | 1(1.4)  18(25.0)  30(41.7)  15(20.8)  8(11.1) | <0.001 |
| Understand relevant genetic information | Not at all confident  A little confident  Confident  More confident  Very Confident | 147(54.4)  92(34.1)  20(7.4)  7(2.6)  4(1.5) | 28(47.5)  24(40.6)  6(10.2)  0  1(1.7) | 4(8.0)  25(50.0)  11(22.0)  8(16.0)  2(4.0) | 23(51.1)  16(35.6)  3(6.6)  3(6.7)  0 | 0  7(29.2)  10(41.7)  5(20.8)  2(8.3) | 51(49.0)  40(38.5)  9(8.7)  3(2.8)  1(1.0) | 4(5.4)  32(43.2)  21(28.4)  13(17.6)  4(5.4) | <0.001 |
| Provide genetic education and mentoring to your peers | Not at all confident  A little confident  Confident  More confident  Very Confident | 207(77.0)  42(15.6)  12(4.5)  3(1.1)  5(1.8) | 36(61.0)  16(27.1)  6(10.2)  0  1(1.7) | 8(16.0)  22(44.0)  10(20.0)  8(16.0)  2(4.0) | 32(69.5)  12(26.1)  1(2.2)  1(2.2)  0 | 0  9(39.1)  10(43.6)  3(13.0)  1(4.3) | 68(64.7)  28(26.6)  7(6.7)  1(1.0)  1(1.0) | 8(11.0)  31(42.4)  20(27.4)  11(15.1)  3(4.1) | <0.001 |
| Advocate for access to desired genetic services | Not at all confident  A little confident  Confident  More confident  Very Confident | 153(58.4)  61(23.3)  28(10.6)  12(4.6)  8(3.1) | 29(50.0)  14(24.1)  12(20.8)  1(1.7)  2(3.4) | 3(6.4)  11(23.4)  20(42.6)  6(12.8)  7(14.8) | 21(46.6)  16(35.6)  7(15.6)  1(2.2)  0 | 0  4(16.6)  10(41.7)  7(29.2)  3(12.5) | 50(48.5)  30(29.1)  19(18.4)  2(2.0)  2(2.0) | 3(4.2)  15(21.1)  30(42.3)  13(18.3)  10(14.1) | <0.001 |
| Assure patients about the confidentiality of the information they provide about family history or their genetic information | Not at all confident  A little confident  Confident  More confident  Very Confident | 96(36.4)  54(20.5)  52(19.7)  20(7.6)  42(15.8) | 12(20.3)  7(11.9)  20(33.9)  7(11.9)  13(22.0) | 2(4.1)  5(10.2)  18(36.7)  9(18.4)  15(30.6) | 13(29.5)  13(29.5)  7(16.0)  3(6.8)  8(18.2) | 0  2(9.1)  9(40.9)  6(27.3)  5(22.7) | 25(24.3)  20(19.4)  27(26.2)  10(9.7)  21(20.4) | 2(2.8)  7(9.9)  27(38.0)  15(21.1)  20(28.2) | <0.001 |
| Recognise the ethical issues associated with taking a family history or genetic testing | Not at all confident  A little confident  Confident  More confident  Very Confident | 97(36.2)  92(34.3)  52(19.4)  10(3.8)  17(6.3) | 15(25.9)  17(29.3)  16(27.6)  6(10.3)  4(6.9) | 1(2.0)  10(20.0)  17(34.0)  12(24.0)  10(20.0) | 11(24.4)  19(42.2)  8(17.8)  3(6.7)  4(8.9) | 0  1(4.3)  10(43.5)  10(43.5)  2(8.7) | 26(25.2)  36(35.0)  24(23.3)  9(8.7)  8(7.8) | 1(1.4)  11(15.1)  27(37.0)  22(30.1)  12(16.4) | <0.001 |
| Incorporate genetic information into routine care | Not at all confident  A little confident  Confident  More confident  Very Confident | 150(56.2)  80(30.0)  25(9.4)  4(1.4)  8(3.0) | 22(37.3)  25(42.4)  9(15.3)  2(3.4)  1(1.6) | 1(2.1)  24(50.0)  12(25.0)  8(16.6)  3(6.3) | 29(64.5)  10(22.2)  5(11.1)  1(2.2)  0 | 0  8(34.8)  8(34.8)  7(30.4)  0 | 51(49.0)  35(33.7)  14(13.5)  3(2.8)  1(1.0) | 1(1.4)  32(45.1)  20(28.2)  15(21.1)  3(4.2) | <0.001 |
| Identify new developments in genetics | Not at all confident  A little confident  Confident  More confident  Very Confident | 195(72.2)  54(20.0)  13(4.8)  3(1.1)  5(1.9) | 32(54.2)  20(33.9)  6(10.2)  0  1(1.7) | 3(6.0)  26(52.0)  14(28.0)  4(8.0)  3(6.0) | 32(69.5)  12(26.1)  1(2.2)  1(2.2)  0 | 1(4.2)  11(45.8)  6(25.0)  6(25.0)  0 | 64(61.0)  32(30.4)  7(6.6)  1(1.0)  1(1.0) | 4(5.4)  37(50.0)  20(27.0)  10(13.5)  3(4.1) | <0.001 |
| Identify gaps in your knowledge about genetics | Not at all confident  A little confident  Confident  More confident  Very Confident | 90(33.4)  63(23.3)  57(21.1)  30(11.1)  30(11.1) | 12(20.3)  13(22.0)  19(32.2)  8(13.6)  7(11.9) | 0  4(8.0)  20(40.0)  15(30.0)  11(22.0) | 12(26.1)  16(34.8)  13(28.3)  2(4.3)  3(6.5) | 0  3(12.5)  7(29.2)  11(45.8)  3(12.5) | 24(22.9)  29(27.6)  32(30.5)  10(9.5)  10(9.5) | 0  7(9.5)  27(36.5)  26(35.1)  14(18.9) | <0.001 |
| Participate in the conduct of genetic-related research | Not at all confident  A little confident  Confident  More confident  Very Confident | 152(56.9)  59(22.1)  37(13.9)  7(2.6)  12(4.5) | 23(39.7)  21(36.2)  8(13.8)  4(6.9)  2(3.4) | 2(4.2)  21(43.7)  9(18.7)  9(18.8)  7(14.6) | 28(60.8)  13(28.3)  4(8.7)  1(2.2)  0 | 0  8(36.3)  8(36.4)  4(18.2)  2(9.1) | 51(49.0)  34(32.7)  12(11.5)  5(4.8)  2(2.0) | 2(2.8)  29(41.4)  17(24.3)  13(18.6)  9(12.9) | <0.001 |
| Participate in genetic-related professional development activities | Not at all confident  A little confident  Confident  More confident  Very Confident | 116(42.9)  65(24.1)  55(20.4)  18(6.7)  16(5.9) | 14(25.4)  20(36.4)  16(29.1)  5(9.1)  0 | 1(2.5)  7(17.5)  20(50.0)  12(30.0)  0 | 18(41.0)  17(38.6)  7(15.9)  2(4.5)  0 | 0  2(10.0)  13(65.0)  5(25.0)  0 | 32(32.3)  37(37.4)  23(23.2)  7(7.1)  0 | 1(1.5)  11(17.0)  34(52.3)  19(29.2)  0 | <0.001 |

*McNemar statistic was used to compare the paired proportions of ‘not at all confident’ versus the combined alternative responses for each item representing ‘some level of confidence’. The calculation only includes the nurses and midwives for whom both pre and post survey data were available.

**Table 5: Learning needs in applying knowledge of genomics and related procedures to practice**

| **Survey Question** |  | | **Pre-workshop Development** | **Workshop 1 and 2**  **(In-Person)** | | **Workshop 3 (Online)** | | **All Workshop Participants** | | ***p* value*** |
| --- | --- | --- | --- | --- | --- | --- | --- | --- | --- | --- |
|  |  | |  | **Pre** | **Post** | **Pre** | **Post** | **Pre** | **Post** |  |
| **Please indicate your education experience and learning needs in the following areas:** | | | n=242 | n=59 | n=49 | n=46 | n=23 | n=105 | n=72 |  |
| Genetics knowledge | | Have learnt about and want more  Have learnt about and do not want more  Have not learnt about but want to  Have not learnt about and do not want to | 95(39.3)  1 (0.4)  143(59.1)  3(1.2) | 33(55.9)  1(1.7)  25(42.4)  0 | 43(87.8)  3(6.1)  3(6.1)  0 | 22(47.8)  0  21(45.7)  3(6.5) | 21(91.4)  1(4.3)  1(4.3)  0 | 55(52.4)  1(1.0)  46(43.8)  3(2.8) | 64(88.8)  4(5.6)  4(5.6)  0 | 0.127 |
| Genetic testing and technologies | | Have learnt about and want more  Have learnt about and do not want more  Have not learnt about but want to  Have not learnt about and do not want to | 71(29.3)  1(0.4)  165(68.2)  5(2.1) | 26(44.9)  1(1.7)  31(53.4)  0 | 41(85.4)  4(8.3)  3(6.3)  0 | 18(39.1)  0  25(54.3)  3(6.6) | 18(78.3)  3(13.0)  2(8.7)  0 | 44(42.3)  1(1.0)  56(53.8)  3(2.9) | 59(83.1)  7(9.9)  5(7.0)  0 | 0.021 |
| Screening and assessment for genetic needs | | Have learnt about and want more  Have learnt about and do not want more  Have not learnt about but want to  Have not learnt about and do not want to | 65(27.0)  2(0.8)  171(71.0)  3(1.2) | 23(40.4)  1(1.8)  33(57.8)  0 | 42(87.4)  2(4.2)  2(4.2)  2(4.2) | 16(34.8)  0  28(60.9)  2(4.3) | 19(82.7)  3(13.0)  1(4.3)  0 | 39(37.9)  1(1.0)  61(59.2)  2(1.9) | 61(86.0)  5(7.0)  3(4.2)  2(2.8) | 0.005 |
| Facilitation of genetic testing | | Have learnt about and want more  Have learnt about and do not want more  Have not learnt about but want to  Have not learnt about and do not want to | 51(22.4)  1(0.4)  169(74.1)  7(3.1) | 23(40.4)  1(1.8)  33(57.8)  0 | 41(87.2)  2(4.3)  4(8.5)  0 | 14(30.4)  0  29(63.0)  3(6.6) | 17(74.0)  5(21.7)  1(4.3)  0 | 37(35.9)  1(1.0)  62(60.2)  3(2.9) | 58(82.9)  7(10.0)  5(7.1)  0 | 0.004 |
| Interpretation of genetic testing | | Have learnt about and want more  Have learnt about and do not want more  Have not learnt about but want to  Have not learnt about and do not want to | 47(20.3)  1(0.5)  176(76.2)  7(3.0) | 23(39.0)  1(1.7)  34(57.6)  1(1.7) | 41(87.2)  3(6.4)  3(6.4)  0 | 13(28.3)  1(2.2)  29(63.0)  3(6.5) | 19(82.6)  2(8.7)  2(8.7)  0 | 36(34.3)  2(1.9)  63(60.0)  4(3.8) | 60(85.8)  5(7.1)  5(7.1)  0 | <0.001 |
| Patient education and communication related to genetics | | Have learnt about and want more  Have learnt about and do not want more  Have not learnt about but want to  Have not learnt about and do not want to | 58(24.4)  1(0.4)  175(73.5)  4(1.7) | 22(38.6)  1(1.8)  34(59.6)  0 | 41(85.4)  2(4.2)  4(8.3)  1(2.1) | 14(31.1)  1(2.3)  28(62.2)  2(4.4) | 18(78.3)  3(13.0)  2(8.7)  0 | 36(35.3)  2(2.0)  62(60.7)  2(2.0) | 59(83.1)  5(7.0)  6(8.5)  1(1.4) | <0.001 |
| Ethical, legal, and social implications of genetic testing | | Have learnt about and want more  Have learnt about and do not want more  Have not learnt about but want to  Have not learnt about and do not want to | 64(26.6)  1(0.4)  172(71.4)  4(1.6) | 24(40.7)  3(5.1)  32(54.2)  0 | 41(83.7)  4(8.2)  3(6.1)  1(2.0) | 16(34.8)  0  27(58.7)  3(6.5) | 18(78.3)  3(13.0)  2(8.7)  0 | 40(38.1)  3(2.8)  59(56.2)  3(2.9) | 59(82.0)  7(9.7)  5(6.9)  1(1.4) | 0.005 |
| Provision of supportive care related to genetic testing | | Have learnt about and want more  Have learnt about and do not want more  Have not learnt about but want to  Have not learnt about and do not want to | 54(23.2)  0  174(74.7)  5(2.1) | 21(35.6)  1(1.7)  37(62.7)  0 | 40(83.3)  3(6.3)  4(8.3)  1(2.1) | 14(31.1)  1(2.2)  26(57.8)  4(8.9) | 18(78.3)  2(8.7)  3(13.0)  0 | 35(33.7)  2(1.9)  63(60.6)  4(3.8) | 58(81.7)  5(7.0)  7(9.9)  1(1.4) | 0.002 |

*McNemar statistic was used to compare the paired proportions of ‘Have learnt about and want more’ versus the combined alternative responses for each item representing ‘other’. The calculation only includes the nurses and midwives for whom both pre and post survey data were available.

**Table 6: Standards for Quality Improvement Reporting Excellence for Education (SQUIRE-EDU)**

| **Title and Abstract** | |  |  |
| --- | --- | --- | --- |
| **1.  Title** | Indicate that the manuscript concerns an [initiative](http://www.squire-statement.org/index.cfm?fuseaction=page.viewpage&pageid=485#Initiative) to improve healthcare (broadly defined to include the quality, safety, effectiveness, patient-centeredness, timeliness, cost, efficiency, and equity of healthcare) | **EDU 1:** Indicate that the manuscript concerns efforts to improve health professions education systems and learning | Pg 1 |
| **2.  Abstract** | a.  Provide adequate information to aid in searching and indexing  b.  Summarize all key information from various sections of the text using the abstract format of the intended publication or a structured summary such as: background, local [problem](http://www.squire-statement.org/index.cfm?fuseaction=page.viewpage&pageid=485#Problem), methods, interventions, results, conclusions | **EDU 2:**Keywords include a focus on education and learning | Pg 2 |
| **Introduction** | *Why did you start?* |  |  |
| [**3. Problem Description**](http://www.squire-statement.org/index.cfm?fuseaction=page.viewpage&pageid=485#Problem) | Nature and significance of the local [problem](http://www.squire-statement.org/index.cfm?fuseaction=page.viewpage&pageid=485#Problem) | **EDU 3**: Description of the nature and significance of the need for change in the local educational system | Pg 3 |
| **4. Available Knowledge** | Summary of what is currently known about the [problem](http://www.squire-statement.org/index.cfm?fuseaction=page.viewpage&pageid=485#Problem), including relevant previous studies |  | Pg 3 |
| **5. Rationale** | Informal or formal frameworks, models, concepts, and/or [theories](http://www.squire-statement.org/index.cfm?fuseaction=page.viewpage&pageid=485#Theory) used to explain the [problem](http://www.squire-statement.org/index.cfm?fuseaction=page.viewpage&pageid=485#Problem), any reasons or [assumptions](http://squire.citysoft.org/index.cfm?fuseaction=page.viewPage&pageID=485&nodeID=1#assumptions) that were used to develop the [intervention(s),](http://www.squire-statement.org/index.cfm?fuseaction=page.viewpage&pageid=485#Interventions)and reasons why the [intervention(s)](http://www.squire-statement.org/index.cfm?fuseaction=page.viewpage&pageid=485#Interventions) was expected to work | **EDU 5**: Identify the guiding theory (learning, change, implementation, or other) and how it aligns with the need for change in the local educational system | Pg 4 |
| **6. Specific Aims** | Purpose of the project and of this report |  | Pg 3 |
| **Methods** | *What did you do?* |  |  |
| [**7. Context**](http://www.squire-statement.org/index.cfm?fuseaction=page.viewpage&pageid=485#context) | Contextual elements considered important at the outset of introducing the [intervention(s)](http://www.squire-statement.org/index.cfm?fuseaction=page.viewpage&pageid=485#Interventions) | **EDU 7a:** Contextual elements for learning (*e.g.*, setting, program, people, resources, social, geopolitical influences) before the intervention(s)  **EDU 7b:** The interrelationships between the contextual elements and the local educational and healthcare systems before the intervention(s) | Pg 3 - Pg 4 |
| [**8. Intervention(s)**](http://www.squire-statement.org/index.cfm?fuseaction=page.viewpage&pageid=485#Interventions) | a.  Description of the [intervention(s)](http://www.squire-statement.org/index.cfm?fuseaction=page.viewpage&pageid=485#Interventions) in sufficient detail that others could reproduce it  b.  Specifics of the team involved in the work | **EDU 8a:** Description of the primary interventions and co-interventions (e.g., faculty or tool development)  **EDU 8b:** Specify how the interprofessional education team (e.g., faculty, staff, patients, and learners) was part of the design of the intervention | Pg 3 – Pg 5 |
| **9. Study of the Intervention(s)** | a.  Approach chosen for assessing the impact of the [intervention(s)](http://www.squire-statement.org/index.cfm?fuseaction=page.viewpage&pageid=485#Interventions)  b.  Approach used to establish whether the observed outcomes were due to the [intervention(s)](http://www.squire-statement.org/index.cfm?fuseaction=page.viewpage&pageid=485#Interventions) | **EDU 9a:**Approach used to understand the impact of the educational intervention(s) on the learner and beyond, such as impact on patients, families, the community, faculty, educational program, or the healthcare system  **EDU 9b**: Approach to assess the fidelity of and the iterative changes to the planned intervention(s) over time | Pg 5 |
| **10. Measures** | a.  Measures chosen for studying [processes](http://www.squire-statement.org/index.cfm?fuseaction=page.viewpage&pageid=485#Process) and outcomes of the [intervention(s),](http://www.squire-statement.org/index.cfm?fuseaction=page.viewpage&pageid=485#Interventions)including rationale for choosing them, their operational definitions, and their validity and reliability  b.  Description of the approach to the ongoing assessment of contextual elements that contributed to the success, failure, efficiency, and cost  c.  Methods employed for assessing completeness and accuracy of data | **EDU 10:** Quantitative and/or qualitative measures chosen to assess the educational processes and outcomes on learners, faculty, educational programs, patients, families, healthcare systems, or communities | Pg 5 |
| **11. Analysis** | a.  Qualitative and quantitative methods used to draw [inferences](http://www.squire-statement.org/index.cfm?fuseaction=page.viewpage&pageid=485#Inferences) from the data  b.  Methods for understanding variation within the data, including the effects of time as a variable |  | Pg 5 |
| **12. Ethical Considerations** | [Ethical aspects](http://www.squire-statement.org/index.cfm?fuseaction=page.viewpage&pageid=485#Ethical_aspects)of implementing and studying the [intervention(s)](http://www.squire-statement.org/index.cfm?fuseaction=page.viewpage&pageid=485#Interventions) and how they were addressed, including, but not limited to, formal ethics review and potential conflict(s) of interest | **EDU 12**: Approaches to address vulnerability of learner participants | Pg 3 |
| **Results** | *What did you find?* |  |  |
| **13. Results** | a.  Initial steps of the [intervention(s)](http://www.squire-statement.org/index.cfm?fuseaction=page.viewpage&pageid=485#Interventions) and their evolution over time (e.g., time-line diagram, flow chart, or table), including modifications made to the intervention during the project  b.  Details of the [process](http://www.squire-statement.org/index.cfm?fuseaction=page.viewpage&pageid=485#Process) measures and outcome  c.  Contextual elements that interacted with the [intervention(s)](http://www.squire-statement.org/index.cfm?fuseaction=page.viewpage&pageid=485#Interventions)  d.  Observed associations between outcomes, interventions, and relevant contextual elements   e.  Unintended consequences such as unexpected benefits, [problems](http://www.squire-statement.org/index.cfm?fuseaction=page.viewpage&pageid=485#Problem), failures, or costs associated with the [intervention(s).](http://www.squire-statement.org/index.cfm?fuseaction=page.viewpage&pageid=485#Interventions)  f.  Details about missing data | **EDU 13a**: For each educational intervention and co-intervention, provide details about iterative modifications based on the assessment of the learning | Pg 5 - Pg 9 |
| **Discussion** | *What does it mean?* |  |  |
| **14. Summary** | a.  Key findings, including relevance to the [rationale](http://www.squire-statement.org/index.cfm?fuseaction=page.viewpage&pageid=485#Rationale) and specific aims  b.  Particular strengths of the project | **EDU 14:**Connect the findings to the guiding theory (learning, change, implementation, other) used to direct the change in the local educational system | Pg 9 - Pg 12 |
| **15. Interpretation** | a.  Nature of the association between the [intervention(s)](http://www.squire-statement.org/index.cfm?fuseaction=page.viewpage&pageid=485#Interventions) and the outcomes  b.  Comparison of results with findings from other publications  c.  Impact of the project on people and [systems](http://www.squire-statement.org/index.cfm?fuseaction=page.viewpage&pageid=485#Systems)  d.  Reasons for any differences between observed and anticipated outcomes, including the influence of [context](http://www.squire-statement.org/index.cfm?fuseaction=page.viewpage&pageid=485#context)  e.  Costs and strategic trade-offs, including [opportunity costs](http://www.squire-statement.org/index.cfm?fuseaction=page.viewpage&pageid=485#Opportunity_costs) | **EDU 15c:** Include the impact of the intervention(s) on learners, faculty, educational program, patients, families, healthcare systems, or communities | Pg 9 – Pg 12 |
| **16. Limitations** | a.  Limits to the [generalizability](http://www.squire-statement.org/index.cfm?fuseaction=page.viewpage&pageid=485#Generalizability) of the work  b.  Factors that might have limited [internal validity](http://www.squire-statement.org/index.cfm?fuseaction=page.viewpage&pageid=485#Internal_validity) such as confounding, bias, or imprecision in the design, methods, measurement, or analysis  c.  Efforts made to minimize and adjust for limitations |  | Pg 9 – Pg 12 |
| **17. Conclusions** | a.  Usefulness of the work  b.  Sustainability  c.  Potential for spread to other [contexts](http://www.squire-statement.org/index.cfm?fuseaction=page.viewpage&pageid=485#context)  d.  Implications for practice and for further study in the field   e.  Suggested next steps | **EDU 17b:** Scalability of the work to other learners and contexts  **EDU 17d:** Lessons learned for clinical practice, education, and policy | Pg 12 |
| **Other Information** | | |  |
| **18. Funding** | Sources of funding that supported this work. Role, if any, of the funding organization in the design, implementation, interpretation, and reporting |  | Pg 1 |
